# Compositional and Temperature Effects on the Rheological Properties of Polyelectrolyte–Surfactant Hydrogels

**DOI:** 10.3390/polym11050927

**Published:** 2019-05-27

**Authors:** Jiří Smilek, Sabína Jarábková, Tomáš Velcer, Miloslav Pekař

**Affiliations:** Faculty of Chemistry, Brno University of Technology, Purkynova 464/118, 612 00 Brno, Czech Republic; xcjarabkova@fch.vut.cz (S.J.); xcvelcer@fch.vut.cz (T.V.); pekar@fch.vut.cz (M.P.)

**Keywords:** Hyaluronan, carbethopendecinium bromide, sodium dodecyl sulphate, diethylaminoethyl-dextran hydrochloride, rheology, hydrogels

## Abstract

The rheological properties of hydrogels prepared by physical interactions between oppositely charged polyelectrolyte and surfactant in micellar form were studied. Specifically, hyaluronan was employed as a negatively charged polyelectrolyte and Septonex (carbethopendecinium bromide) as a cationic surfactant. Amino-modified dextran was used as a positively charged polyelectrolyte interacting with sodium dodecylsulphate as an anionic surfactant. The effects of the preparation method, surfactant concentration, ionic strength (the concentration of NaCl background electrolyte), pH (buffers), multivalent cations, and elevated temperature on the properties were investigated. The formation of gels required an optimum ionic strength (set by the NaCl solution), ranging from 0.15–0.3 M regardless of the type of hydrogel system and surfactant concentration. The other compositional effects and the effect of temperature were dependent on the polyelectrolyte type or its molecular weight. General differences between the behaviour of hyaluronan-based and cationized dextran-based materials were attributed to differences in the chain conformations of the two biopolymers and in the accessibility of their charged groups.

## 1. Introduction

Interactions between polyelectrolytes and oppositely charged surfactants constitute an area of intensive research not only for theoretical reasons but also because of the practical applications of these systems, e.g., in cosmetic, pharmaceutical, and food products [1]. In this work the interactions of polyelectrolytes with surfactants in micellar form are of special interest. Kizilay et al. [1] overviewed the mechanistic and structural features of the complexation and coacervation of polyelectrolytes with oppositely charged colloids, including surfactant micelles. The first interaction step is the linking of the colloid and polyelectrolyte, which is described by two types of model—either the “condensation” of polyelectrolyte chains on the surface of colloidal particles or the binding of colloids as “ligands” to host polyelectrolytes. Generally, polyelectrolyte–colloid interactions depend mainly on the charge per polyelectrolyte repeat unit, the ionic strength, pH and the colloid charge density. The effect of the polyelectrolyte molecular weight is usually negligible [1]. Association typically continues in several subsequent steps, controlled also by the concentration of interacting species: non-interacting, individual polymers and colloids (e.g., due to the low concentration of interacting species or the subcritical colloid surface charge density); primary complexes of an intrapolymer type; soluble aggregates; and coacervates (the whole system is separated into two immiscible liquid phases, one of which—the coacervate—is relatively concentrated in macromolecules). Intrapolymer complexes are formed by individual polymer chains decorated with colloids and have radii similar to those of the maternal polyelectrolyte. Soluble aggregates are formed by the association of primary complexes. Coacervation arises from extended interactions among soluble aggregates (complexes) and is considered to be a true form of liquid–liquid phase separation. In many systems, maximum coacervation is found when the charges of the colloid and polyelectrolyte are neutralized [1]. However, particularly in polyelectrolyte–micelle systems, the coacervation region is broader than the electroneutrality point. Entropic effects, related to counter ion release or the formation of partially charged neutralized droplets also play a role in interactions between oppositely charged polyelectrolyte-colloids [1].

At surfactant concentrations well above the critical micelle concentration, interactions with oppositely charged polyelectrolyte can lead to the separation of a gel-like (viscoelastic) material. Kizilay et al. [2] attributed the viscoelastic properties of coacervate to a “disproportionation” process, during which poly-ions and their counter ions in systems where the poly-ion charge exceeds that of colloids are expelled to domains of 50–300 nm in size. Thus, proximal regions of more complete charge neutralization and higher density are created, which serve as crosslinks in the resulting viscoelastic material. The formation of these domains is driven by similar forces such as the formation of neutral aggregates in solution—the enthalpic contribution of interacting opposite charges—and the entropic contribution from both counter ion release and chain configuration change.

Polyelectrolyte–surfactant gels retain the amphiphilic character of the surfactant building blocks—inside the hydrophilic gel matrix they contain hydrophobic micelle-like domains capable of solubilizing non-polar species [2]. However, these materials are still underexplored and only limited information on their properties can be found in the literature. Perhaps the most information can be found on hyaluronan–cationic surfactant systems. Thalberg and Lindman [3] describe the separation of a few volume percent of a highly viscous gel-like phase after mixing hyaluronan with decyl-, dodecyl, tetradecyl- or cetyltrimethylammonium bromide. Just the gel phase formed by hyaluronan and tetradecyltrimethylammonium bromide was studied by Wong et al. using NMR techniques [4]. They observed that, in the gels, the surfactant molecules formed relatively small micellar aggregates which were bound to the polyelectrolyte chains. The surfactant aggregates seemed to be completely covered with polyelectrolyte chains at polyelectrolyte/surfactant ratios above 0.5 (by weight). Thalberg and Lindman [5] prepared the gels from hyaluronan of various molecular weights and decyl-, dodecyl, or tetradecyltrimethylammonium bromide. The formation of the gel phase was observed for the hyaluronan molecular weight below about 20,000 g·mol^−1^. No crystalline phases were detected with small-angle X-ray scattering. The gels were able to solubilize a hydrophobic dye (in contrast to hyaluronan–water systems); thus they should contain hydrophobic domains. Proton NMR relaxation revealed the presence of micellar aggregates in the gels. Analogical self-diffusion studies showed that surfactants in micellar solutions had self-diffusion coefficients of the same order of magnitude as those measured in the gels. The surfactant self-diffusion was attributed both to the diffusion of its monomers within the micellar aggregates and to the monomer exchange between different aggregates. Water self-diffusion was reduced in the gels by obstruction and the presence of hydration water within the gel structure.

Buchold et al. [6] investigated hyaluronan–tetradecyltrimethyl ammonium bromide systems at high surfactant concentrations and in the presence of 160 mM NaBr. They also presented a phase diagram covering several decades of surfactant concentration in which a two-phase area can be observed. The authors reported that one of the phases was a turbid, dense gel which was not formed under salt-free conditions, a finding which is consistent with our previous study [7]. However, only single phase solutions with very high surfactant concentrations (245 mM and more) were further investigated in that study [8].

Recently, we reported on the rheological properties of hyaluronan–cetyltrimethylammonium bromide hydrogels and demonstrated how they can be controlled over a broad range of liquid-like to solid-like states by the polyelectrolyte molecular weight [7]. In this work, we focused in more detail on the effects of various processing parameters on the properties of similar gels prepared using a slightly different cationic surfactant (approved for use in pharmaceutical formulations) and also investigated a reversely charged system—a positively charged polyelectrolyte (cationized dextran) and an anionic surfactant.

## 2. Materials and Methods

The sodium form of hyaluronan (HYA) was purchased from Contipro (Dolní Dobrouč, Czech Republic) and used as anionic polyelectrolyte without further treatment. In this study, two types of hyaluronan were used, high and low molecular weight hyaluronan (HMW and LMW, respectively). Diethylaminoethyl-dextran hydrochloride (Sigma-Aldrich, Prague, Czech Republic, batch BCBQ8681 with 3.0% of nitrogen; DEAED) was used as received as the cationic polyelectrolyte. The exact molecular weights of all polysaccharides were checked by the SEC-MALLS technique and the results are shown in Table S1 (Supplementary Material).

Carbethopendecinium bromide (Septonex, Czech Pharmacopoeia quality), a pharmaceutical cationic surfactant, was purchased from GBNchem (Prague, Czech Republic). Sodium dodecyl sulphate (SDS, ≥99.0%), purchased from Sigma Aldrich (Czech Republic), and was used as received as the anionic surfactant.

The composition of the gel samples was selected on the basis of previous experience and preliminary experiments. Basically, when increasing the concentration of polyelectrolyte at a constant surfactant concentration, or vice versa, the gel started to form at a certain concentration, then its amount increased up to a maximum, after which it decreased upon a further increase in surfactant concentration. Therefore, we selected concentrations at which sufficient amounts of gel were formed and at which the viscosity of the polyelectrolyte solutions was not too high to complicate the mixing and preparation of the samples. The surfactant concentration should be well above its critical micelle concentration (cf. Table 1).

Hydrogels were prepared in two ways—the solution or powder method. The solution method was based on the simple mixing of the pre-prepared surfactant and polyelectrolyte solutions with the required concentrations. We found that gels could also be prepared by pouring the liquid solvent over the mixture of powdered polyelectrolyte and surfactant; this was called the powder method, which is patent pending [9].

The solution method was realized by mixing a stock solution of polysaccharide and a stock solution of surfactant in a volume ratio of 1:1 in a vial and leaving the vial on a shaker overnight to complete the gelation process and the separation of the gel phase. Stock solutions were prepared in 0.15 M NaCl using deionized water (Purelab Flex, ELGA system), because previous experiments showed that a non-zero ionic strength of the aqueous medium is important for obtaining gel-like materials [7]. The initial concentrations of stock solutions are listed in Table 1. The theoretical charge ratio was calculated supposing the existence of one charge on each surfactant molecule, one charge per hyaluronan basic dimeric unit (sodium form) with a molecular weight of 401.299 g·mol^−1^, and one charge per each nitrogen atom in DEAED.

In the powder method, required amounts of biopolymer and surfactant in powder form were weighed directly into the vials. Then, an appropriate volume of 0.15 M NaCl aqueous solution was added. The final concentrations of the resulting mixtures were half of those listed in Table 1. The samples were vortexed briefly and left overnight to complete the gelation process and phase separation.

Alternatively, phase separation in both methods was accelerated by centrifugation. The polysaccharide and surfactant stock solutions were thoroughly mixed in a centrifuge tube and centrifuged at 4000 rpm for 10 min.

The effect of multivalent ions was investigated by preparing samples by the powder method and using corresponding salt solutions (CaCl_2_, MgCl_2_ and FeCl_3_) at a concentration of 0.15 M (instead of NaCl). Similarly, the effect of pH was studied using appropriate buffer solutions of ionic strength 0.15 M. The composition of buffer solutions is summarized in Table S2. In addition, samples in the study of the effect of ionic strength and pH were prepared by the powder method using aqueous solutions of NaCl at different concentrations (0.05–1.0 M).

Rheological measurements were performed on an AR-G2 rheometer (TA Instruments, New Castle, DE, USA) using steel plate-plate geometry (a diameter of 25 or 8 mm, gap size of 100 μm). Experiments were carried out at least in duplicates, under a controlled temperature of 25 or 37 °C. Each sample was equilibrated for 3 min at the given temperature before measurement (conditioning). The maximum normal force used for compressing the sample did not exceed 5 N. During measurement, a solvent trap was used to prevent water evaporation and subsequent changes in the hydrogel structure. First, the linear viscoelastic region (LVR) was determined by strain sweep tests (deformation of 0.01–1000%, frequency of 1 Hz, 6 points per decade). Viscoelastic properties (elastic and viscous moduli, complex viscosity) as a function of oscillation frequency were then determined by frequency sweep tests in the range of 0.01–20 Hz, the deformation chosen within the LVR (the chosen amplitude of deformation was the same for all frequency sweeps). Steady state shear experiments were performed in the shear rate range of 0.02–200 s^−1^ (logarithmic sweep, 6 points per decade). Each experiment was conducted at least twice. The results are the average of all measurements. The standard deviation did not exceed 7% in either case—frequency or flow measurement.

The fundamental rheological parameters such as relaxation moduli (G) and relaxation time (λ) were calculated from oscillatory measurements with respect to the Maxwell model [10]. For each hydrogel, the mesh size was calculated from the frequency sweep measurements according to Equation (1) [10]

(1) $$\xi =\sqrt[{-3}]{\frac{G_{\infty }}{k_{B}T}}$$

where ξ represents the mesh size calculated in [m], $G_{\infty }$ is the value of the storage modulus when the plateau has been reached (or, if the plateau is not reached, the value of the storage modulus at the maximum frequency of oscillation), $k_{B}$ is the Boltzmann constant, and *T* represents the absolute temperature.

An alternative way of determining the mesh size was also tested. The mesh size was calculated according to Pescosolido et al. [11] On the basis of measurements and rheological investigation, it was concluded that the optimal number of Maxwell elements corresponds to four in order to fit the hydrogel viscoelastic curves. According to this finding, it is possible to calculate the hydrogel shear modulus as the sum of all relaxation moduli.

The mechanical properties of polyelectrolyte–surfactant hydrogels can be analysed using rubber elasticity theory [12]. The application of this theory on biopolymer hydrogels has been questioned. Nevertheless, recent studies [13] have shown that for hydrogels with elastic components, such as the polyelectrolyte–surfactant hydrogels described, this theory can be applied. One of the most important assumptions is that the mechanical properties of hydrogels are determined in the linear viscoelastic region (the amplitude of deformation in frequency sweep tests must always be chosen from the linear viscoelastic region). If this condition is met, a new parameter—crosslink density ($\rho_{x}$)—can be calculated according to Equation (2) [11].

(2) $$\rho_{x}=\frac{G}{RT}$$

where *R* is the universal gas constant, *T* represents the absolute temperature, and *G* is the shear modulus. From the crosslink density, which can give us information about the density of the junction (unit: mol·m^−3^), the mesh size can be calculated with respect to Equation (3) [11].

(3) $$\xi =\sqrt[{3}]{\frac{6}{\pi \rho_{x}N_{A}}}$$

## 3. Results and Discussion

### 3.1. The Effect of the Preparation Method

Hydrogels of the same composition were prepared by different techniques, as described in Materials and methods. First, the solution and powder methods were compared. It was found that both methods gave materials with very similar rheological properties in both oscillatory and shear tests, which indicates that a final, equilibrium state was reached. An example can be found in Figure S1 (Supporting material), where the frequency sweep for H1 is shown. The independency of viscoelastic properties on the preparation method was observed for all tested hyaluronan and dextran hydrogels. This fact is important also from the point of view of potential applications—it would not be necessary to prepare a solution but just to mix powders and the liquid dispersion medium directly, which would be more cost-effective.

The separation of hydrogels by free standing, i.e., by gravitation, was rather time consuming (the phase preparation of hydrogels took at least 24 h), which is undesirable with respect to potential commercial production. Therefore, the preparation methods were modified by centrifuging the system after mixing the components. The accelerated separation of the gels did not affect their rheological properties (data not shown).

Thus, the various preparation methods did not affect the mechanical properties of the resulting hydrogels. For further investigations, gel samples were separated by the centrifugation method.

### 3.2. The Effect of Surfactant Concentration

#### 3.2.1. Oscillatory Tests

Strain sweep measurements primarily give information on the extent of the linear viscoelasticity region, but can also be used to detect the effects of composition. Generally, the surfactant concentration demonstrated a very small effect on the shape of the strain sweep curves and corresponding moduli (an example is given in Figure S2 in Supplementary Material).

The storage modulus of HMW hyaluronan-based gels was higher than the loss modulus throughout the whole linear viscoelasticity region, stressing the gel-like character of all materials. The highest *G*′/*G*″ ratio was found for intermediate surfactant concentrations (samples H2, H5), which indicated the increased rigidity of densely crosslinked materials. In contrast, the loss modulus of LMW hyaluronan-based gels was higher than the storage modulus. The length of the linear viscoelasticity region of hyaluronan-based gels was dependent on the surfactant concentration—interestingly, the length increased with the concentration of surfactant for HMW hydrogels and decreased for LMW hydrogels (cf. Table S3 in Supplementary Material). Thus, the hyaluronan molecular weight influenced the structural destruction of the gels—longer chains are necessary to prepare materials with enhanced resistance to the mechanical destruction of their networks. The lowest value of the storage modulus in the linear viscoelasticity region was found for sample H3, where the charge ratio was 1:1. This means that micelles could be bound both in an electrostatic and steric manner in an excess of surfactants in the case of samples H1 and H2, which possessed the most rigid behaviour.

Hydrogels prepared from DEAED and a lower concentration of SDS (sample D2) exhibited higher absolute values of both moduli (elastic and viscous). The linear viscoelastic region was shorter for the D1 sample. Viscous moduli exceeded elastic moduli during the whole of each measurement and the difference between elastic and viscous moduli was the same for both concentrations of SDS (the example is given in Figure S3 in Supplementary Material).

The frequency sweep is the main method of oscillatory rheometry. The effect of surfactant concentration on frequency sweep results was dependent on the hyaluronan molecular weight. Gels prepared from HMW hyaluronan showed similar shapes of the measured curves (see the example on Figure S4 in Supplementary Material). Also, the modulus value at the crossover point was only weakly dependent on the surfactant concentration. However, the crossover frequency was shifted to higher values with increasing surfactant concentration, i.e., the corresponding relaxation time decreased in the same time (Table S4). The calculated mesh size slightly decreased when the surfactant concentration increased and was generally smaller for HMW hyaluronan gels. The smaller mesh size for HMW hyaluronan gels can be explained by the more dense coiled structure of HMW hyaluronan in comparison with LMW biopolymer, which is subsequently crosslinked by surfactant micelles [14] (see Table 2).

The sample of HMW hyaluronan gels with the lowest concentration of surfactant (H3) had theoretically saturated all potential sites of electrostatic interactions (the charge ratio was 1). Of course, this supposes the total dissociation and accessibility of all charged groups, which, in real samples, will not likely be attained [6]. Nevertheless, at an increased surfactant concentration, at least some micelles are not bound electrostatically but physically entrapped in the gel network and function like a (nano-sized) filler. This could also explain why the viscoelastic properties change slightly with an increasing concentration of surfactant and why the mesh size is slightly decreased with an increasing concentration of surfactant.

In the case of LMW hyaluronan, almost the same general change in moduli with surfactant concentration as in HMW hyaluronan-based hydrogels was observed. However, due to the shorter chain length, gels with predominantly elastic moduli could be prepared only when using the highest Septonex concentration (200 mM), which means that excess surfactant (micelles), can participate in the network as an elastically active component. Lower concentrations of Septonex (samples H5, H6) caused the loss moduli of hydrogels to be higher over the whole range of frequencies. Materials formed from LMW hyaluronan generally behaved more like a viscoelastic liquid than a (soft) solid.

The values of the principal measured or calculated parameters are given in Table 2. Discrete relaxation spectra were obtained with five elements (see Table S4)—the relaxation spectra for HWM hyaluronan hydrogels practically overlapped and showed a plateau during the first three or four relaxation times followed by a noticeable decrease (Figure S5). In the case of LMW hyaluronan hydrogels, relaxation moduli decreased significantly with increasing relaxation time. This decrease was practically independent of the Septonex concentration.

**Figure 1:**
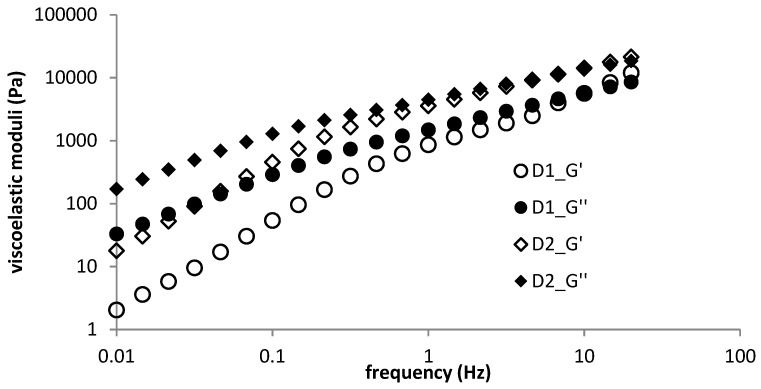
Frequency sweep for diethylaminoethyldextran hydrochoride (DEAE) hydrogels—concentration dependence.

In the case of DEAED-based hydrogels, increased surfactant concentration significantly changed their rheological behaviour. For samples with lower surfactant concentrations, both viscoelastic moduli were up to one order of magnitude higher than in the case of samples with higher surfactant concentrations; the crossover point was shifted to a somewhat lower frequency with a higher value of crossover modulus (Table 2). Higher moduli values for samples with a lower concentration of surfactant mean tougher hydrogels (Figure 1).

The gels prepared with higher surfactant concentrations also demonstrated a much lower slope in their relaxation spectra (Figure S6 in Supplementary Material), i.e., slower relaxation. In this case, the excess of surfactant functioned much more like a “lubricant” than a reinforcing filler, causing a deterioration in mechanical properties.

#### 3.2.2. Flow Curves

All hydrogels were sufficiently soft to be subject to flow curve measurements. In contrast to oscillatory tests, much bigger deformations are employed in these tests and the network structure is thus definitely disturbed. Generally, using an oscillatory technique, the structure of hydrogels should not be damaged within the linear viscoelasticity region.

All hydrogels demonstrated pseudoplastic behaviour with a Newtonian plateau at low shear rates. An increased surfactant concentration in hyaluronan-based gels resulted in increased zero-shear viscosity (see Table S5 in Supplementary Material); i.e., micelles worked as thickening fillers. LMW hyaluronan hydrogels had lower viscosities than their HMW counterparts, which is attributable to their shorter chain lengths [15,16]. Their HMW counterparts also possessed a much shorter Newtonian plateau and a steeper viscosity decrease after this plateau. DEAED-based hydrogels manifested increased zero-shear viscosity at the lower surfactant concentration, which is consistent with findings in oscillatory tests, whereas the width of the Newtonian region was weakly dependent on the surfactant concentration (Table S5 in Supplementary Material).

The observed pseudoplastic behaviour was a result of relatively weak bonding interactions, which could be relatively easy disrupted by low shear rates. These physical interactions were destroyed at high shear rates and the biopolymer chains in tattered hydrogels were oriented in the direction of flow, decreasing the apparent viscosity.

**Figure 2:**
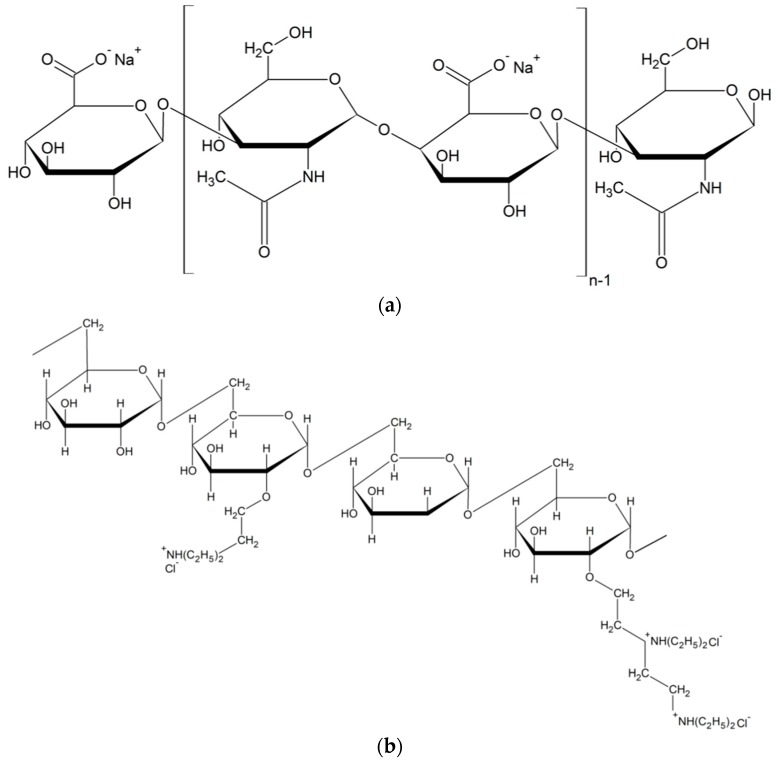
(**a**) Structure of sodium hyaluronate; (**b**) structure of modified dextran (DEAE—diethylaminoethyldextran hydrochoride).

The differences between the rheological behaviour of hyaluronan- and DEAED-based gels may be attributed to the structural differences in their polysaccharide backbone [17]. Hyaluronan probably possesses more opportunities for hydrogen bonding and forms a single strain alpha helix [18], whereas dextran adopts a ribbon like conformation with two antiparallel chains [19,20,21,22]. Further, there should be differences in the accessibility of the charged groups amenable to electrostatic interactions. Whereas the positively charged groups protrude from the dextran chain, the negatively charged carboxyls on hyaluronan are positioned much closer to its backbone—see the structures in Figure 2. The impact of such accessibility on polyelectrolyte–surfactant interactions was reported by Buchold et al. [6].

### 3.3. Effect of Temperature

The viscoelastic properties of biopolymer–surfactant hydrogels were determined at 25 and 37 °C. These temperatures were chosen purposefully, because of the potential dermal applications of these hydrogels. Increased temperature had a very small effect on the strain sweep curves of both LMW and HMW hyaluronan-based hydrogels. The linear viscoelasticity region remained almost unchanged, and moduli very slightly increased with increased temperature in all hydrogels prepared from hyaluronan (for an example see Figure S7 in Supplementary Material).

In the case of DEAED-based hydrogels, the temperature effect on the moduli was rather stronger, while the linear viscoelasticity region also remained almost unchanged for both hydrogels with different concentrations of SDS (an example is shown in Figure S8 in Supplementary Material). Both viscoelastic moduli were slightly shifted to higher values at the elevated temperature.

**Figure 3:**
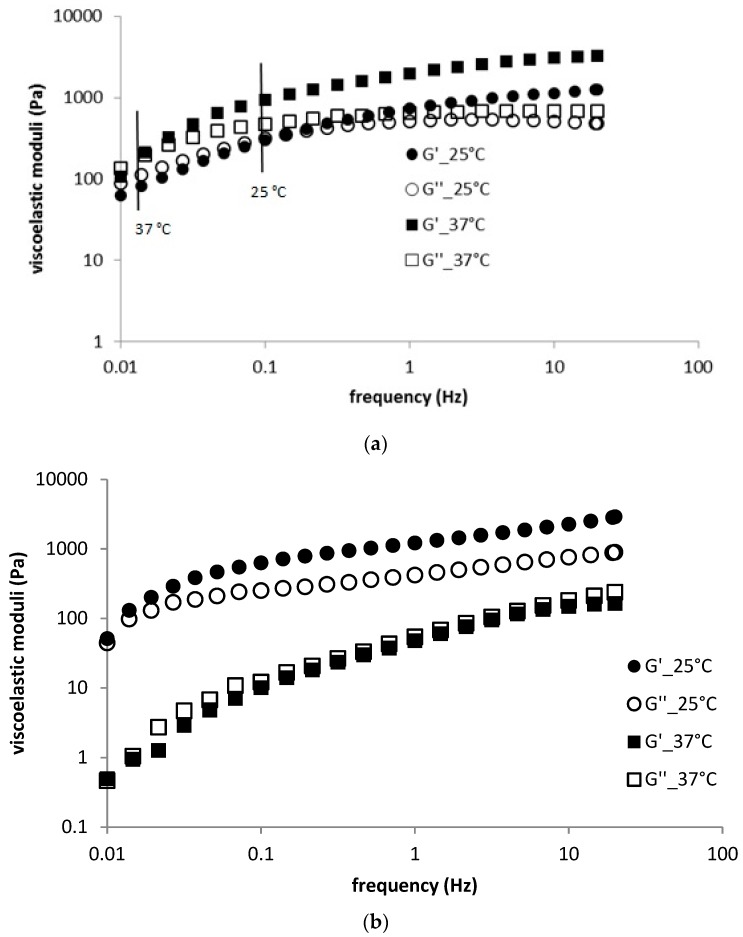
(**a**) Frequency sweep for H1 sample—temperature dependence; (**b**) frequency sweep for H4 sample—temperature dependence

The temperature effect on frequency sweep measurements was dependent on the hyaluronan molecular weight. In the case of HMW Ha, temperature increased the values of both moduli (more distinctly, the elastic modulus) and shifted the crossover point significantly to lower frequencies; also, the crossover modulus value was decreased (an example is shown in Figure 3a). The relaxation spectrum remained almost unchanged when the temperature increased (see Figure S9 in Supplementary Material). Thus, to some extent, a higher temperature stiffened the structure of the hydrogel prepared from HMW hyaluronan.

In contrast, increased temperature significantly reduced the moduli of LMW hyaluronan-based hydrogels and both moduli exhibited very similar values at the higher temperature over the whole frequency range (an example is given in Figure 3b). The crossover point seemed to remain essentially unchanged from the point of view of frequency, whereas the crossover modulus value was probably significantly lower at the elevated temperature (determining the exact crossover location is problematic due to the almost completely overlapping corresponding curves). The relaxation spectrum was shifted downwards and to the left with temperature (cf. the example in Figure S9 in Supplementary Material).

The polymer molecular weight is thus crucial for the heat resistance of the structure and elasticity of hyaluronan polyelectrolyte–surfactant hydrogels. Short chains form a looser network structure which is released by a moderate increase in temperature. In contrast, the structure formed by long chains is stiffened, which suggests the accessibility of more crosslinking points at the elevated temperature, probably as a result of the higher molecular mobility and conformational rearrangements of biopolymer chains.

**Figure 4:**
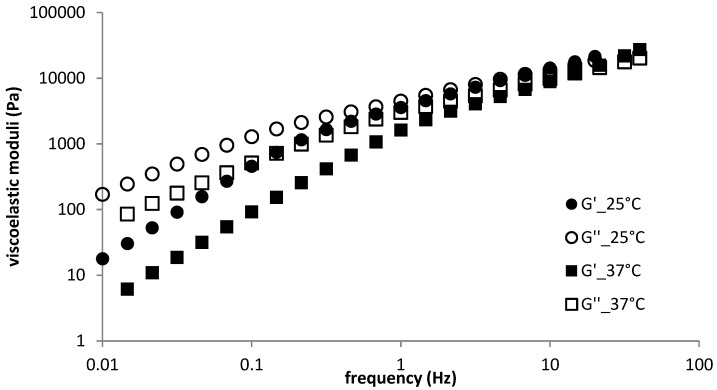
Frequency sweep for sample D2—temperature dependence.

The temperature effect on the frequency sweep of DEAED–SDS hydrogels (at both concentrations of SDS) was manifested by decreased moduli values and increased crossover frequencies at the elevated temperature (example is shown in Figure 4). The crossover modulus value was not substantially changed by changes in temperature. The relaxation spectrum was shifted downwards with increased temperature for both systems (see the example in Figure S10 in Supplementary Material). The behaviour at the elevated temperature was thus similar to that of LMW hyaluronan-based gels.

Flow curves were not significantly affected by temperature in the case of HMW hyaluronan-based hydrogels and DEAED–SDS hydrogels. The disturbed network structure of the resulting fluid-like materials was thus not sensitive to the applied temperature increase. LMW hyaluronan-containing materials demonstrated increased viscosity and no Newtonian plateau at the higher temperature, as shown in Figure S11 in Supplementary Material.

### 3.4. Effect of Ionic Strength

The viscoelastic properties of HMW hyaluronan-based hydrogels were strongly dependent on ionic strength. Hydrogels with lower ionic strength (0.05 M NaCl) had a higher elastic modulus than viscous modulus at higher frequencies of oscillation. The crossover point was shifted to higher frequencies in comparison with a standard sample (0.15 M NaCl); moreover, both moduli exhibited lower absolute values. On the other hand, at a higher ionic strength (0.3 M NaCl), both moduli were distinctly shifted to lower absolute values in comparison with standard samples or hydrogels with a lower ionic strength. The viscous modulus exceeded the elastic modulus over the whole range of measurable frequencies, which means that materials with a higher ionic strength behave more liquid-like. Moreover, hydrogels at yet higher ionic strengths (>0.3 M NaCl) could not be prepared—there was no gelation process.

The same behaviour was also observed for LMW hyaluronan-based hydrogels. Furthermore, the critical ionic strength of the LMW hyaluronan-hydrogels gelation process was lower than for HMW hyaluronan-based hydrogels—when an ionic strength of 0.15 M NaCl was exceeded, the gelation process was not observed.

DEAED–SDS-based hydrogels with the highest ionic strength (0.5 M) could not be prepared due to surfactant solubility problems in this environment. Samples with lower (0.05 and 0.1 M) and higher (0.3 and 0.5 M) ionic strengths than the standard ionic strength (0.15 M) were prepared without problems. On the other hand, hydrogel based on DEAED–SDS in pure water without the adjustment of ionic strength could not be prepared. Non-zero ionic strength is required for the preparation of DEAED–SDS hydrogel (at least 0.05 M).

The linear viscoelastic region of DEAED–SDS hydrogels was independent of ionic strength and was comparable with that of the standard sample. The results from frequency sweep test measurements showed a very small effect of ionic strength on sample D2 and a toughening effect of increased ionic strength on sample D1.

Flow curves for DEAED–SDS-based hydrogels detected a very small effect of ionic strength in comparison with flow curves for standard samples. Flow curves at the changed ionic strength were close to the flow curve for the standard sample, especially in the Newtonian region. The biggest difference in flow curves was demonstrated by the hydrogel prepared with an ionic strength of 0.3 M, which had the highest viscosity values in the non-Newtonian region.

Gelation in both biopolymer systems was sensitive to ionic strength. Gels could not be prepared without the addition of low molecular weight electrolyte. Thalberg and Lindman [3] reported that a certain minimum concentration of surfactant is needed for the marked formation of complexes between hyaluronan and cationic surfactants. A too high surfactant concentration is known to suppress polyelectrolyte–surfactant interactions due to the screening of electrostatic forces. The same was observed in this work for DEAED systems. Ions of the added electrolyte affect biopolymer polyelectrolyte conformations and the aggregation of surfactants (by changing their critical micellar concentration and acting as additional counter ions to dissociated surfactant moieties). For the formation of hydrogel materials and achieving adequate rheological properties, there is an optimum ionic strength for both biopolymers, which lies around 0.15–0.3 M.

### 3.5. Effect of pH (Buffers)

Standard gel samples (i.e., samples without extraneous pH control) prepared from hyaluronan were of essentially neutral pH, whereas DEAED-based gels were slightly acidic (see Table 3).

Generally, changes in pH decreased both moduli and suppressed the elastic behaviour of hyaluronan-based hydrogels during strain sweep in comparison to a standard sample. The effect of pH buffers has been studied only for phase-separated hydrogels based on HMW hyaluronan. In the case of LMW hyaluronan, the elastic modulus was lower than the viscous modulus and in some samples even unmeasurable. In the case of HMW hyaluronan, both moduli had comparable values, which decreased with increasing pH, except for the highest pH value where the moduli values were the highest.

No effect of pH was observed on the width of the viscoelastic region in DEAED hydrogels. D1 hydrogels could be prepared only in a pH range of 4.5–7, which is very close to the pH of standard sample; no gels were formed in more acidic or alkaline environments. In contrast, it was possible to prepare D2 hydrogels throughout the chosen pH range. More acidic buffer slightly toughened the D2 hydrogels.

**Figure 5:**
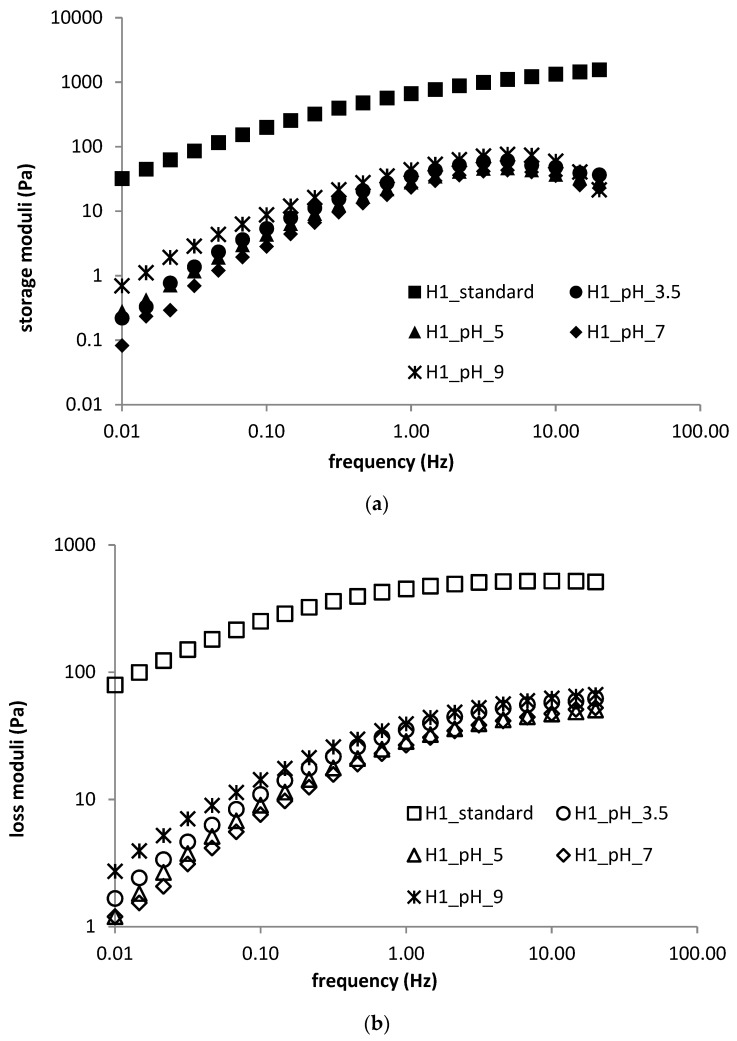
(**a**) Frequency sweep (storage moduli) for H1 sample—pH dependence; (**b**) frequency sweep (loss moduli) for H1 sample—pH dependence

Frequency sweep investigations resulted qualitatively in the same conclusions—LMW hyaluronan resulted in a liquid-like, viscous material with practically unmeasurable elastic moduli (in contrast to the corresponding standard samples). The curves measured for HMW hyaluronan gels were all located about two orders of magnitude below the curves for the standard sample. They were slightly shifted to lower moduli values with increasing pH, except for the highest pH curve, which gave the highest moduli (see Figure 5a,b). The crossover point frequency first increased with increasing pH, then dropped down for the highest pH. The effect of pH on the crossover modulus was small.

The D1 standard sample achieved the lowest viscoelastic moduli in frequency sweep tests. Both moduli thus increased with increased pH and were almost identical for D1 gels prepared in buffers of pH 5 and 7. Crossover moduli and crossover frequencies were shifted to higher and lower values, respectively, in comparison to the standard sample. The effect of pH on D2-based gels was much smaller—decreasing pH somewhat toughened the gels, while the highest pH resulted in gels with the lowest moduli, which were very close to the moduli of the standard sample (see Figure S12). Whereas the crossover modulus was comparable for gels prepared at the three lower pH values, its value dropped two orders of magnitude for the gel prepared at the highest pH; similar behaviour was also observed for the crossover frequency (see Table 4). The effect of pH on the basic parameters of the frequency sweep tests is summarized in Table S7.

**Figure 6:**
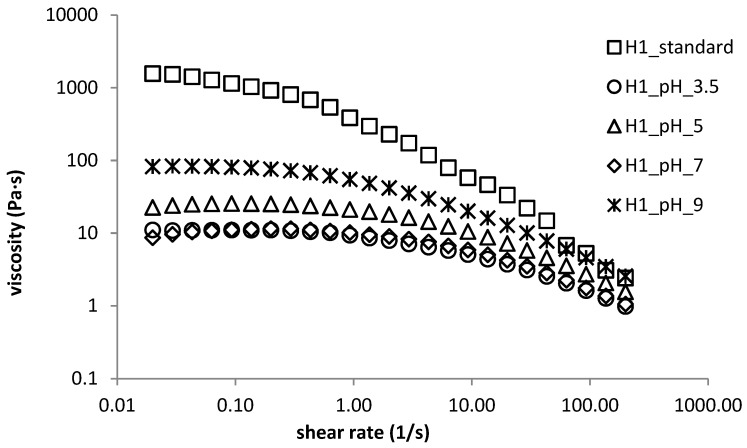
Flow properties of H1 sample—pH dependence.

The effect of pH on flow curves was also dependent on hyaluronan molecular weight. Flow curves measured for HMW hyaluronan-containing gels (see Figure 6) almost always showed lower apparent viscosities than the standard sample. Gels prepared at pH 9 possessed the highest viscosities whereas those prepared at pH 7 had the lowest. All flow curves started to overlap at the highest frequencies. In the case of LMW hyaluronan-containing materials, there was about a one order of magnitude decrease in apparent viscosities and a reduction in the Newtonian region compared with the standard sample. There was no significant effect of pH on the flow properties of hydrogels prepared from DEAED and SDS, which accords with the results of oscillatory tests. All hydrogels, independently of pH, displayed pseudoplastic behaviour, with mostly overlapping flow curves possessing a clear Newtonian plateau at low shear rates up to approximately 1.0 s^−1^, with the exception of the standard sample, where the Newtonian plateau ended at approximately 0.2 s^−1^.

The results obtained with hydrogels prepared in buffer solutions demonstrated that besides the effect of pH the effect of buffer ions should also be considered, despite the fact that the buffers were prepared at identical ionic strengths. Acidic pH should protonate hyaluronan and thus suppress its electrostatic interactions with surfactant micelles and, consequently, the formation of crosslinks. This was confirmed, particularly in the case of LMW hyaluronan-based gels. Samples prepared in phosphate buffer had approximately similar pH in comparison to the standard sample; however, different rheological properties and decreased acidic pH slightly toughened HMW hyaluronan-based hydrogels. Thus, differences in the size and charge of buffer ions seemed to affect the structures of the formed materials. In the case of DEAED-based gels, a much stronger influence of surfactant concentration on the effect of pH was observed. This could be a result of the better accessibility of DEAED charged groups together with the higher sensitivity of anionic surfactant to pH and buffer ions. Much higher sensitivity to the buffer environment was detected in the sample with higher surfactant concentration—both acidic and alkaline environments hindered the formation of gel material. Whereas alkaline conditions should suppress DEAED charging (the dissociation of amino groups), acidic environments should tend to neutralize the surfactant charge. At lower surfactant concentrations, weak gel was already formed in the absence of buffer, which was not very sensitive to the addition of buffer ions.

### 3.6. Effect of Multivalent Ions

The presence of multivalent ions affected the linear viscoelasticity region of hyaluronan-based hydrogels detected in strain sweep experiments. The effect of ions on moduli values was stronger in the case of LMW hyaluronan-based hydrogels. The highest moduli were obtained for hydrogels containing Fe^3+^ ions, the lowest for hydrogels with Ca^2+^ ions (HMW hyaluronan) or Mg^2+^ ions (LMW hyaluronan). All hydrogels containing multivalent ions had lower viscoelastic moduli in comparison with the standard sample (without the addition of multivalent ions), because all added cations had the same charge as the micelles and therefore could supress polymer–micelle interactions. On the other hand, Fe^3+^ ions are known to crosslink hyaluronan chains themselves, which could be the reason for the highest moduli observed among the tested ions [23]. The range of the linear viscoelastic region was independent of the presence of multivalent ions.

The linear viscoelastic region of DEAED gels was weakly dependent on the presence and type of multivalent ions (Ca^2+^ ions had the biggest effect) and was comparable with that of the standard sample without the addition of multivalent ions. The effect of multivalent ions on the viscoelastic properties of DEAED hydrogels was dependent on the surfactant concentration. In the case of D1 samples, the presence of multivalent ions caused an increase in both moduli. The highest increase was observed for Ca^2+^ ions. In contrast, sample D2 containing multivalent ions had lower viscoelastic moduli in comparison with the standard sample. Again, the greatest influence was observed for Ca^2+^ ions (more than one order of magnitude higher than standard sample moduli). In contrast to hyaluronan-based hydrogels, there was no significant influence of ferric ions on the values of viscoelastic moduli.

In DEAED-based samples the added multivalent cations had an opposite charge to the surfactant micelles and were thus supposed to affect the hydrogels’ behaviour mainly through their interactions with micelles. This is in accord with the observed dependence of the effect of the cations on the surfactant concentration. In an excess of surfactant (a charge ratio greater than 1, D1 samples) the added cations were observed to have a stiffening effect, which could be attributed to the formation of new structures between the added cations and micelles which did not participate in the biopolymer–surfactant network crosslinks. In the case of D2 samples, a proportion of the micelles interacted with the added cations, which disturbed the biopolymer–surfactant network and lowered its rheological parameters. (The basic characteristics of the linear viscoelasticity region in the presence of multivalent ions are summarized in Tables S6 and S7 in Supplementary Material).

**Figure 7:**
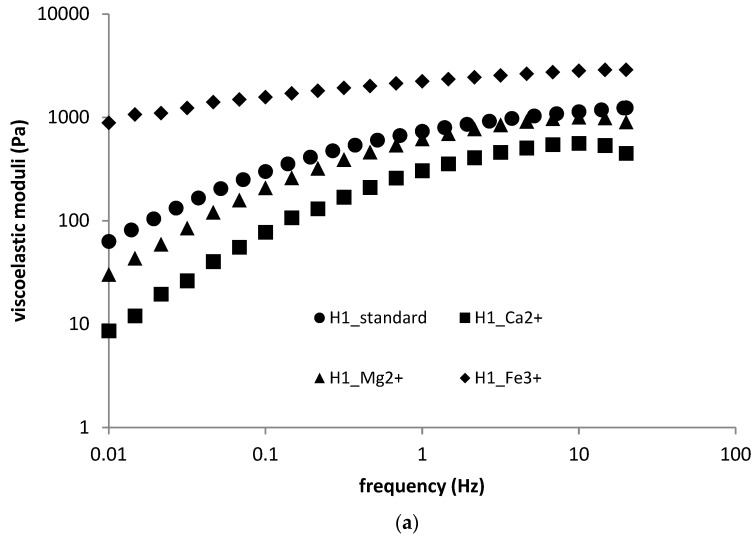

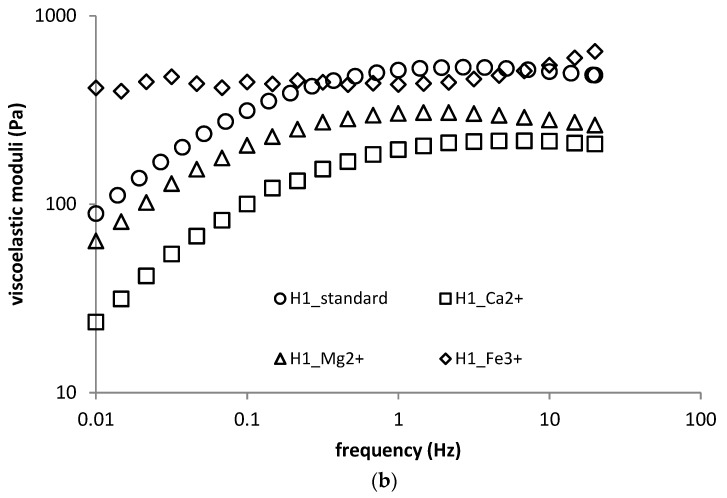
(**a**) Frequency sweep (storage moduli) for H1 sample—the effect of multivalent ions; (**b**) frequency sweep (loss moduli) for H1 sample—the effect of multivalent ions

The frequency sweep of HMW hyaluronan-based gels showed a stiffening effect on the part of Fe^3+^ ions, whereas bivalent ions decreased the values of both moduli (for a representative example, see Figure 7a,b).

In particular, ferric ions increased the storage modulus at lower frequencies and shifted the cross-over point to very low frequencies (in fact, the storage modulus outweighed the loss modulus across the whole of the measured frequency range). The frequency sweep of LMW hyaluronan-based gels possessed the same behaviour as that of HMW hyaluronan-based gels in the environment of bivalent ions, where both moduli were slightly decreased. In the presence of ferric ions, no gelation process was observed. The effect of multivalent ions on the viscoelastic properties of both types of hydrogels is summarized in Table 5.

**Figure 8:**
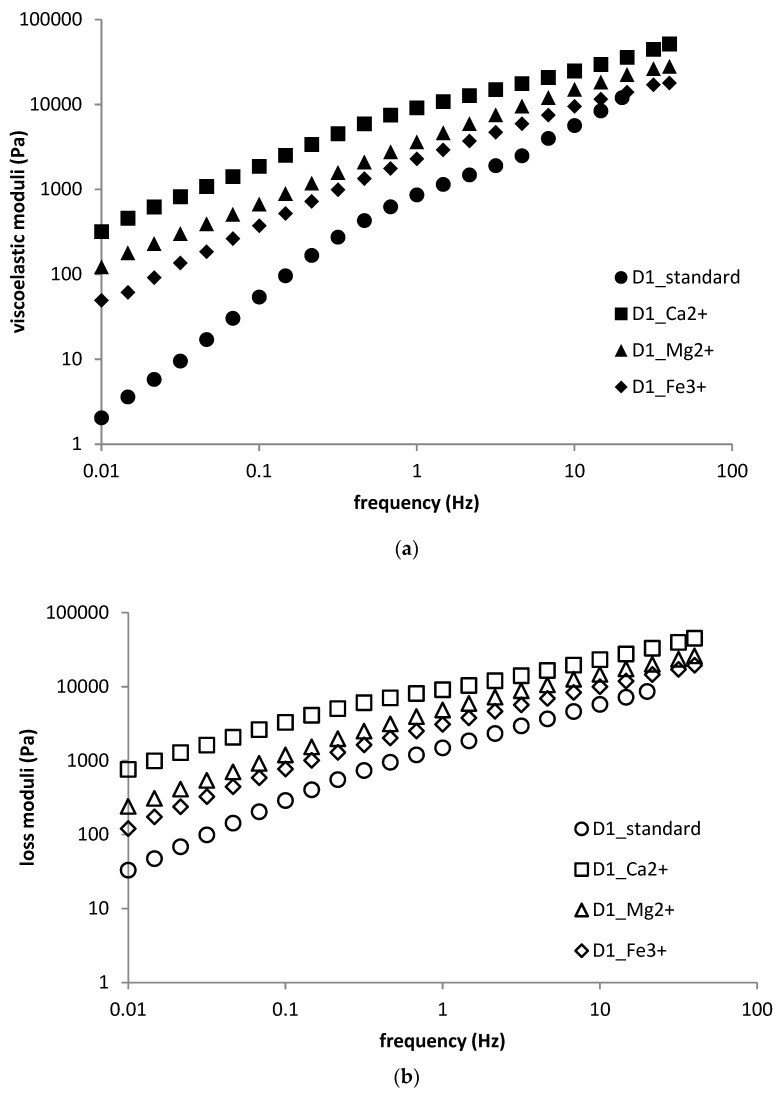
(**a**) Frequency sweep (storage moduli) for D1 sample—the effect of multivalent ions; (**b**) frequency sweep (loss moduli) for D1 sample—the effect of multivalent ions

In the case of DEAED-based hydrogels, the results of frequency sweep investigations basically corresponded to those obtained from strain sweep measurements. Hydrogels with higher surfactant concentrations and containing multivalent ions had lower viscoelastic moduli, whereas those with lower surfactant concentrations had higher viscoelastic moduli in comparison with the standard sample. Hydrogels containing calcium ions had the biggest influence on viscoelastic moduli, which were more than one order of magnitude higher (D1) or lower (D2) than standard sample moduli, see Figure 8a,b.

Flow curves detected a very small effect of bivalent ions on hydrogels prepared from both HMW and LMW hyaluronan (Figures S13 and S14 in Supplementary Material); in the case of LMW biopolymer, the curves were shifted slightly upwards, i.e., the ions slightly increased viscosity. The effect of Fe^3+^ ions was stronger, particularly in the case of LMW hyaluronan, where viscosity increased by up to four orders of magnitude and the increase in viscosity was inversely proportional to the shear rate (Figure S14).

Multivalent ions somewhat increased the apparent viscosity within the Newtonian plateau of the standard sample D1, whereas a decrease was observed in the case of D2 samples (Figures S15 and S16). Also, with respect to D1 samples, the Newtonian plateau was disturbed by multivalent ions in the sense that the viscosity was not constant but slightly decreasing. In the presence of multivalent ions, the viscosity decrease in the non-Newtonian region was not so steep in D1 samples and almost unchanged in D2 samples, except in the Ca-containing sample. Calcium ions had generally the strongest effect on flow curves of DEAED-based hydrogels.

## 4. Conclusions

Hydrogels were prepared from oppositely charged biopolymer polyelectrolyte and surfactant in micellar form at a surfactant:biopolymer charge ratio at least equal to one. Hyaluronan acted as a negatively charged biopolymer, whereas DEAED (amino-modified dextran) was used as a positively charged biopolymer. The former interacted with Septonex (carbethopendecinium bromide) whereas the latter interacted with sodium dodecylsulphate. The rheological properties of hyaluronan-based hydrogels were dependent mainly on the polymer molecular weight. Surfactant concentration (more precisely, the concentration of micelles and a surfactant:biopolymer charge ratio above 1) showed only a small effect. Surfactant concentration was found to have a much greater effect for DEAED-based hydrogels.

Gels were formed at an optimum ionic strength (set by NaCl solution), ranging from 0.15–0.3 M regardless of the type of hydrogel system or surfactant concentration. Outside the optimum range, the formation of either very weak gels (viscoelastic liquids) or viscous liquids was observed.

The effect of increased temperature (37 °C) was dependent on the hydrogel composition and biopolymer (hyaluronan) molecular weight. Both the stiffening and weakening of gels with increased temperature was observed.

Preparing gels in buffers of acidic, neutral, or alkaline pH generally deteriorated their mechanical properties in comparison to hydrogels prepared in NaCl solution with the optimum concentration (ionic strength). The best materials were thus prepared in model physiological solution (0.15 M NaCl) at natural pH. Besides controlling the pH, the buffers also affected the hydrogel properties due to the presence of their ions, which changed the environment of the ionically crosslinked hydrogels.

The addition of multivalent cations decreased the values of the rheological parameters of hyaluronan-based hydrogels probably due to their interference in the interactions between hyaluronan and micelles possessing the same charge as the added cations. In the case of DEAED-based materials, a stiffening effect on the part of the added cations was observed in the presence of excess surfactant charge as a result of their interactions with (excess) micelles which were not involved in the hydrogel network crosslinks.

General differences between the behaviour of hyaluronan and DEAED materials were attributed to the differences in the chain conformations of the two biopolymers and in the accessibility of their charged groups. To sum up our work, it was experimentally proved that the final flow and viscoelastic properties of phase-separated hydrogels based on surfactant:biopolymer can be certainly modulated by changing of chemical composition of the hydrogels as well as physico-chemical parameters (temperature, ionic strength, presence of multivalent ions). These findings are crucial for the potential applications of phase-separated hydrogels on a wider level.

## Figures and Tables

**Table 1:** Concentrations of initial solutions of hyaluronan (HYA), DEAED (Diethylaminoethyl-dextran hydrochloride), Septonex and SDS (Sodium dodecyl sulphate) used to prepare hydrogels by the solution method.

Sample Name	Hyaluronan		DEAED	Septonex	SDS	Charge Ratio
	HMW (High Molecular Weight)	LMW (Low Molecular Weight)				
	% (*w*/*v*)			mM		
H1	2			200		4.0
H2	2			100		2.0
H3	2			50		1.0
H4		2		200		4.0
H5		2		100		2.0
H6		2		50		1.0
D1			4		400	4.7
D2			4		100	1.2

**Table 2:** Cross-over frequency and modulus, mesh size for all prepared hydrogels.

Sample Name	Cross-over Frequency (Hz)	Cross-over Modulus (Pa)	Mesh Size (nm)
H1	0.14	324.5	13.01
H2	0.10	287.8	14.56
H3	0.05	314.2	16.41
H4	0.01	65.6	20.4
H5	4.64	242.2	22.8
H6	2.15	199.1	21.6
D1	10.00	5765	8.94
D2	6.81	11430	7.12

**Table 3:** pH values of hydrogels and supernatants.

Sample Name	pH	
	Gel Phase	Supernatant
H1	6.88	7.09
H2	7.23	7.20
H3	7.15	7.00
H4	6.83	7.32
H5	7.05	7.25
H6	7.38	7.13
D1	4.58	6.74
D2	4.93	6.60

**Table 4:** Cross-over frequency and modulus, mesh size for all prepared hydrogels, pH influence.

pH	Sample Name	Cross-over Frequency (Hz)	Cross-over Modulus (Pa)	Mesh Size (nm)
**3.5**	H1	0.954	34.24	48.25
	H2	1.474	43.19	43.75
	H3	1.732	29.03	56.58
	H4	gel not observed		
	H5	gel not observed		
	H6	gel not observed		
	D1	gel not observed		
	D2	4.511	16320	3.93
**5**	H1	1.175	29.47	52.27
	H2	0.507	55.82	28.71
	H3	1.972	35.57	54.93
	H4	gel not observed		
	H5	gel not observed		
	H6	gel not observed		
	D1	11.660	16440	5.72
	D2	8.055	15220	5.46
**7**	H1	3.160	37.54	56.52
	H2	1.196	40.19	50.00
	H3	2.121	27.02	61.19
	H4	gel not observed		
	H5	gel not observed		
	H6	gel not observed		
	D1	1,468	4250	
	D2	7.853	13740	5.18
**9**	H1	0.650	34.14	57.67
	H2	18.070	78.23	38.34
	H3	1.674	19.79	68.41
	H4	gel not observed		
	H5	gel not observed		
	H6	gel not observed		
	D1	gel not observed		
	D2	0.248	871	6.82

**Table 5:** The effect of multivalent ions to cross-over point in samples, values of frequency and G’ = G".

Sample Name	Cross-over Point							
	Original Sample		CaCl_2_		MgCl_2_·6H_2_O		FeCl_3_	
	Moduli (Pa)	Frequency (Hz)	Moduli (Pa)	Frequency (Hz)	Moduli (Pa)	Frequency (Hz)	Moduli (Pa)	Frequency (Hz)
H1	328	0.14	206	0.16	200	0.09	227	0.01
H2	278	0.10	207	0.07	246	0.14	-	<0.01
H3	338	0.06	255	0.05	154	0.05	-	<0.01
H4	69	0.01	477	19.85	90	0.11	75	0.12
H5	245	4.77	584	6.51	89	0.15	74	0.12
H6	192	2.04	522	2.55	73	0.11	76	0.05
D1	5765	10.00	9089	1.00	14670	10.00	17140	31.62
D2	11430	6.81	no cross-point		5142	3.16	1560	1.47

## Supplementary Materials

The supplementary materials are available online at https://www.mdpi.com/2073-4360/11/5/927/s1.
