# Supplementary material for: Compositional and Temperature Effects on the Rheological Properties of Polyelectrolyte–Surfactant Hydrogels
Source: Polymers (Basel). 2019 May 27;11(5):927. doi: 10.3390/polym11050927 (PMC6571672; doi:10.3390/polym11050927)
Supplement: Supplementary file 1 [file polymers-11-00927-s001.pdf]

polymers

MDPI

Appendix – Supplementary material

Sample	Abbreviation	Molecular weight (kDa)
High molecular weight hyaluronan	HMW HYA	1255 ± 90
Low molecular weight hyaluronan	LMW HYA	309 ± 4
Diethylaminoethyl-dextran hydrochloride	DEAED	729 ± 32

Table S1. Molecular weights of all polysaccharides determined by SEC-MALLS technique

pH range	Used chemicals	Buffer name
3,5 – 5,5	$CH_3COONa \cdot 3\ H_2O$; CH_3COOH	Acetate buffer
6 – 7,5	KH_2PO_4; NaOH	Phosphate buffer
8 – 9	HCl; $Na_2B_4O_7 \cdot 10\ H_2O$	Borate buffer
9,5 – 11	NaOH; $Na_2B_4O_7 \cdot 10\ H_2O$	Borate buffer

Table S2. Substances used for buffers preparation for study of pH effect

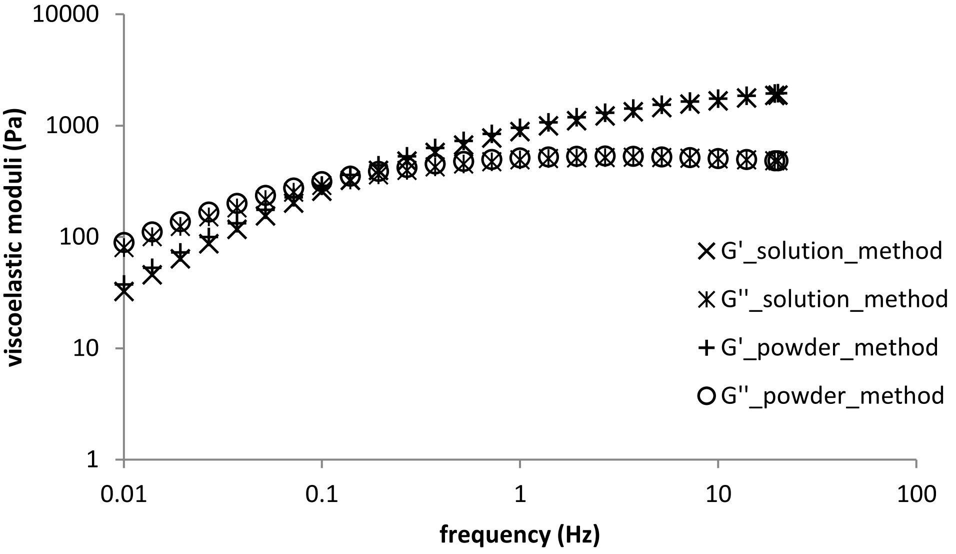

Figure S1. Comparison of viscoelastic properties of H1 sample prepared by different ways (solution vs. powder method)

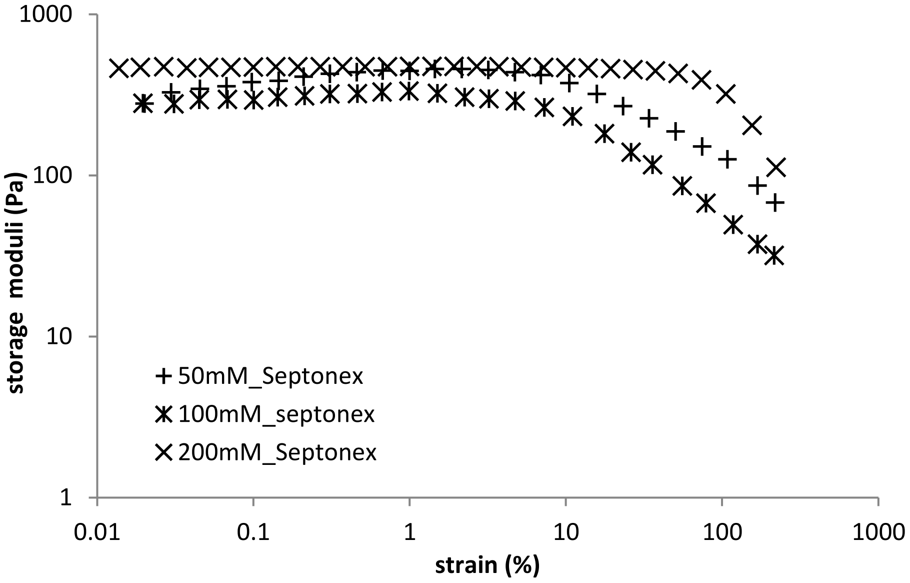

Figure S2. Storage moduli (G′) for HYA hydrogels prepared – concentration dependence

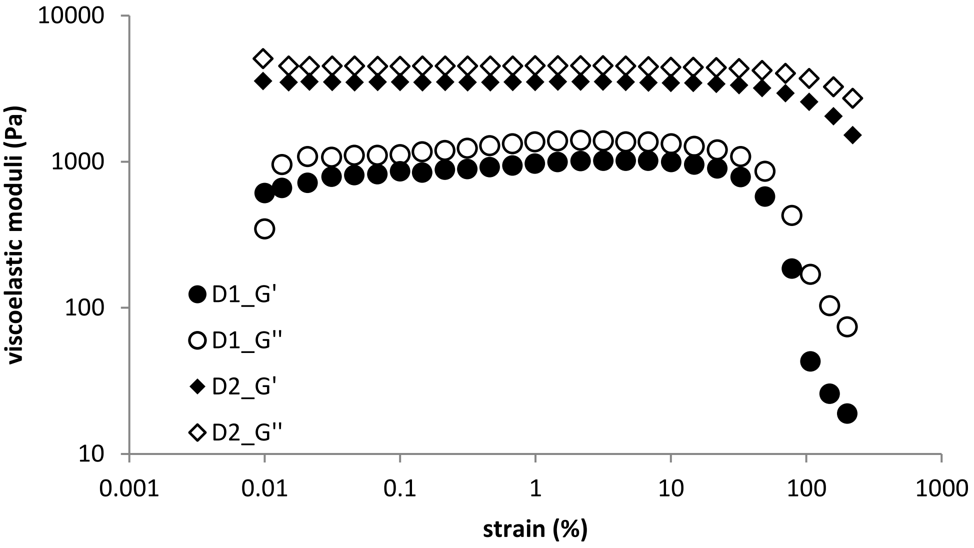

Figure S3. Strain sweep for DEAED hydrogels – concentration dependence

Sample	End of linear viscoelastic region (%)
H1	19.30
H2	7.75
H3	5.91
H4	1.94
H5	4.77
H6	10.18
D1	21.98
D2	70.32

Table S3. The end of linear viscoelastic region range for hydrogels prepared from HMW and LMW hyaluronan

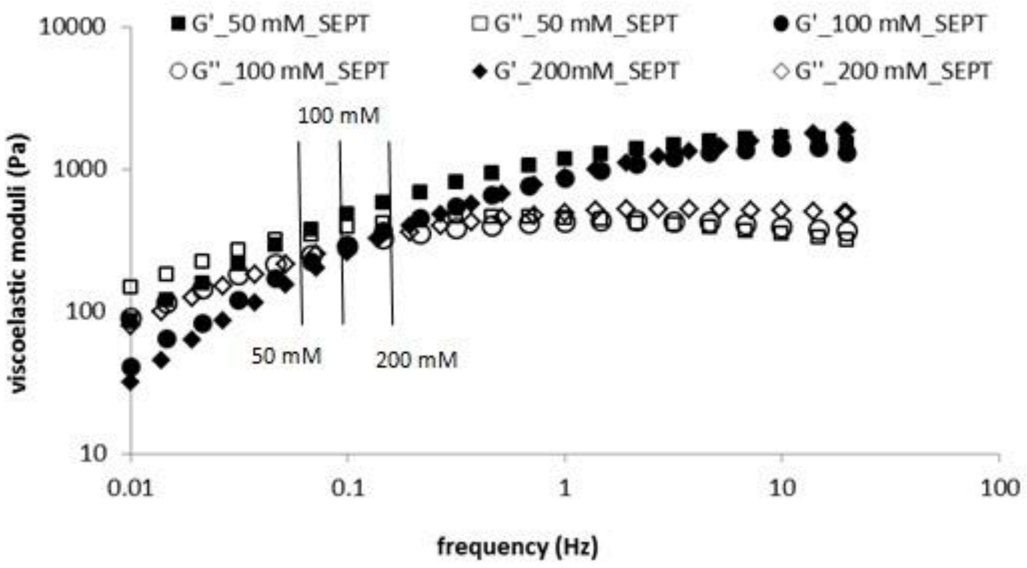

Figure S4. Frequency sweep for H1, H2, H3 samples

**Sample name**	**G_1 (Pa)**	**λ_1 (s)**	**G_2 (Pa)**	**λ_2 (s)**	**G_3 (Pa)**	**λ_3 (s)**	**G_4 (Pa)**	**λ_4 (s)**	**G_5 (Pa)**	**λ_5 (s)**
H1	803.1	0.009	607.2	0.069	509.3	0.352	286.6	1.824	72.2	12.240
H2	588.9	0.010	389.1	0.061	485.0	0.265	354.9	1.586	100.5	11.800
H3	508.7	0.010	435.2	0.072	508.5	0.340	464.3	1.735	194.8	11.830
H4	1576.0	0.010	542.2	0.082	142.8	0.209	78.3	0.780	20.3	1.976
H5	648.0	0.010	170.9	0.056	97.7	0.153	22.5	1.729	12.1	12.350
H6	511.3	0.009	228.0	0.074	72.7	0.456	18.5	1.958	9.6	12.010
D1	12170	0.013	953.1	0.140	471.5	0.478	16.39	1.856	6.262	8.379
D2	31380	0.009	4900	0.093	1954	0.488	365.6	1.872	30.79	11.470

Table S4. Relaxation spectra parameters for all tested hydrogels

Sample name	Zero-shear viscosity (Pa.s)	Shear rate at the end of Newton plateau (1/s)
H1	1499 ± 105	0.03
H2	54 ± 6	0.09
H3	14.3 ± 0.7	0.06
H4	10.8 ± 0.4	0.29
H5	3.29 ± 0.08	2.94
H6	1.12 ± 0.05	0.20
D1	396 ± 8	2.00
D2	2715 ± 212	1.36

Table S5. Zero-rate viscosity and Newtonian plateau for all studied hydrogels

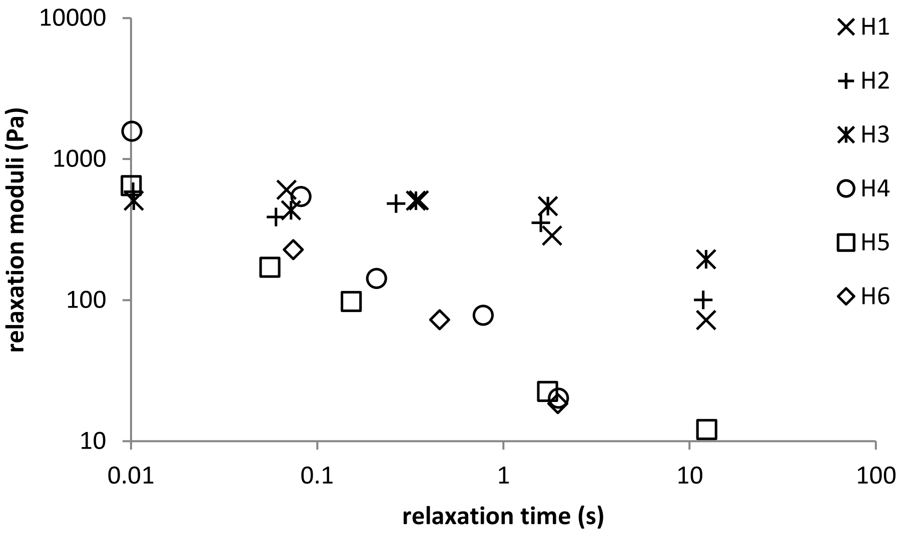

Figure S5. Relaxation spectra for H1-H6 samples

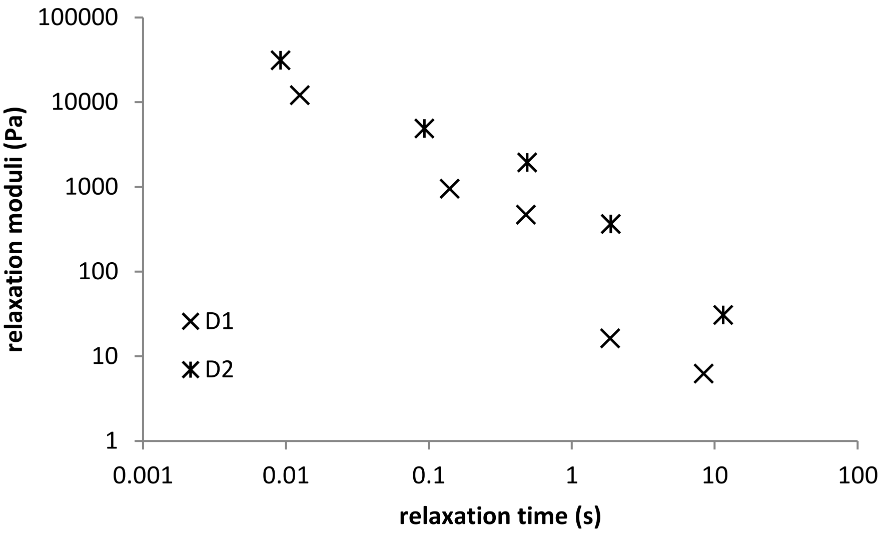

Figure S6. Discrete relaxation spectra for DEAED hydrogels – concentration dependence

39
40

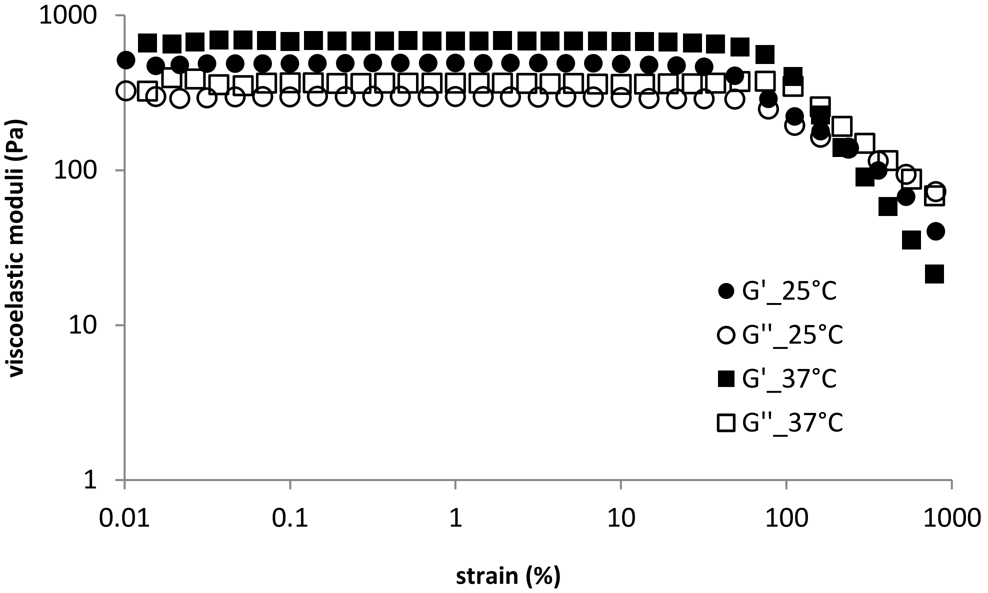

Figure S7. Strain sweep for H1 sample - temperature dependence

41

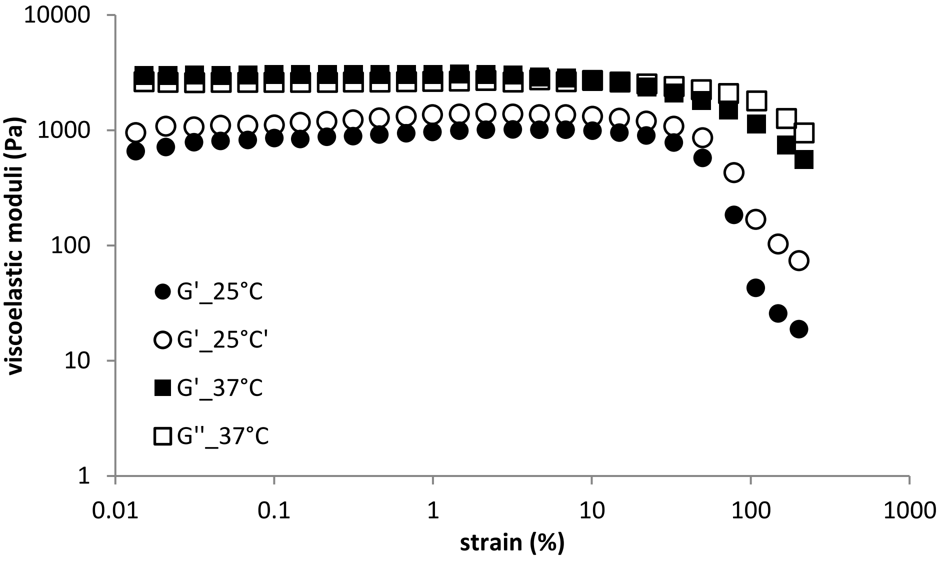

Figure S8. Strain sweep for D1 sample – temperature dependence

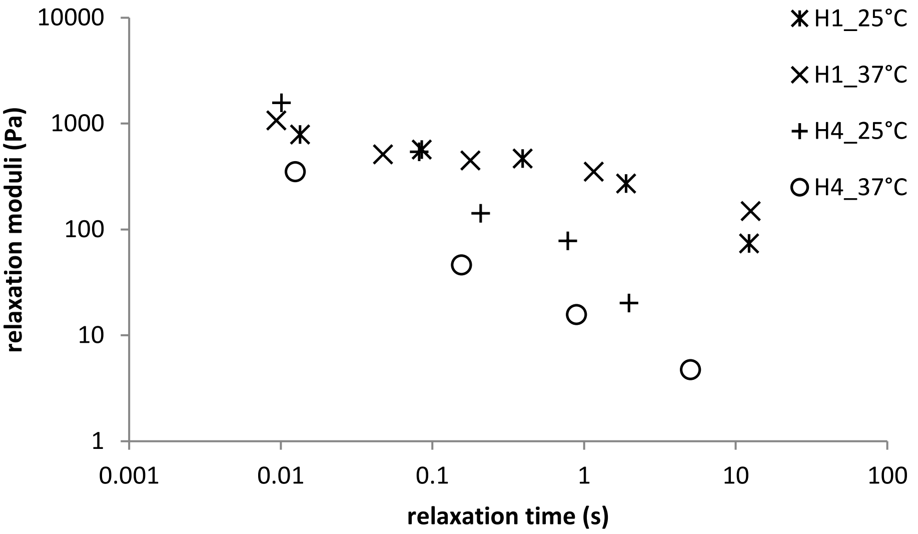

Figure S9. Discrete relaxation spectra for H1 and H4 samples – temperature dependence

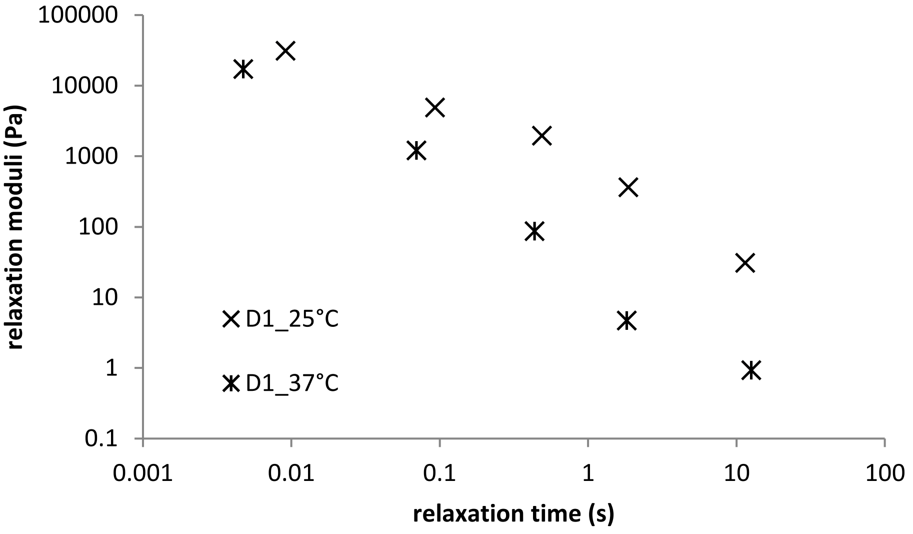

Figure S10. Discrete relaxation spectra for D1 sample – temperature dependence

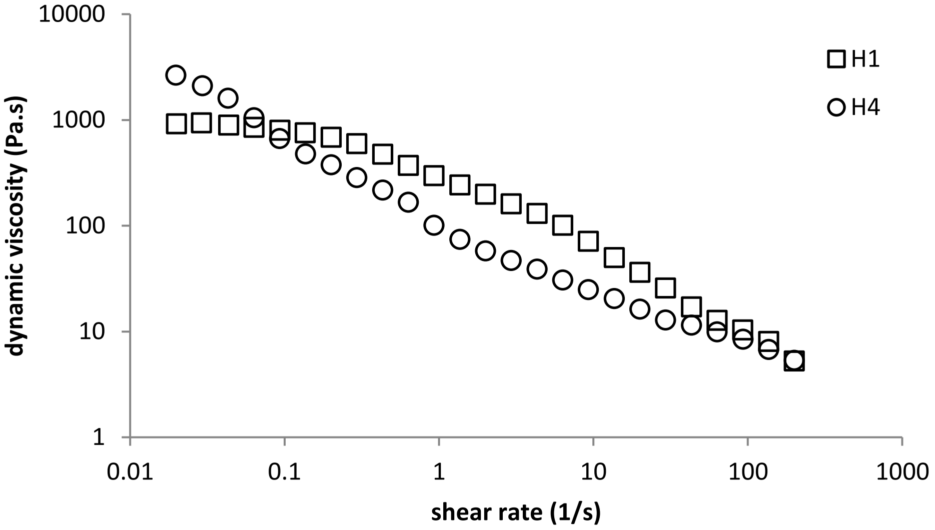

Figure S11. Flow properties of H1 and H4 samples (37 °C)

**Sample**	**Linear viscoelastic region (%)**				**Viscoelastic moduli G′/G″ (Pa)**			
	**$CaCl_2$**	**$MgCl_2\cdot 6H_2O$**	**$FeCl_3$**	**Original samples**	**$CaCl_2$**	**$MgCl_2\cdot 6H_2O$**	**$FeCl_3$**	**Original samples**
**H1**	75.63	75.15	103.59	19.30	277/198	544/377	1163/308	481/268
**H2**	50.83	225.96	32.15	4.75	1414/719	66/64	177/88	289/79
**H3**	29.28	15.66	14.94	6.91	415/201	635/216	300//99	42/13
**H4**	100.71	221.79	No gel	1.94	65/126	30/74	No gel phase detected	210/105
**H5**	149.95	216.91	No gel	4.77	109/199	7.6/30	No gel phase detected	168/36
**H6**	21.63	No gel phase detected	4.69	10.18	169/241	No gel phase detected	321/162	64/19
**D1**	1.51	14.99	10.15	21.98	7544/7949	3321/4664	1075/1625	899/1206
**D2**	1.47	69.86	47.20	70.32	666/737	2216/2553	699/835	2939/4015

Table S6. Linear viscoelastic region ranges for hydrogels. Describe the effect of multivalent ions to range of linear viscoelastic region.

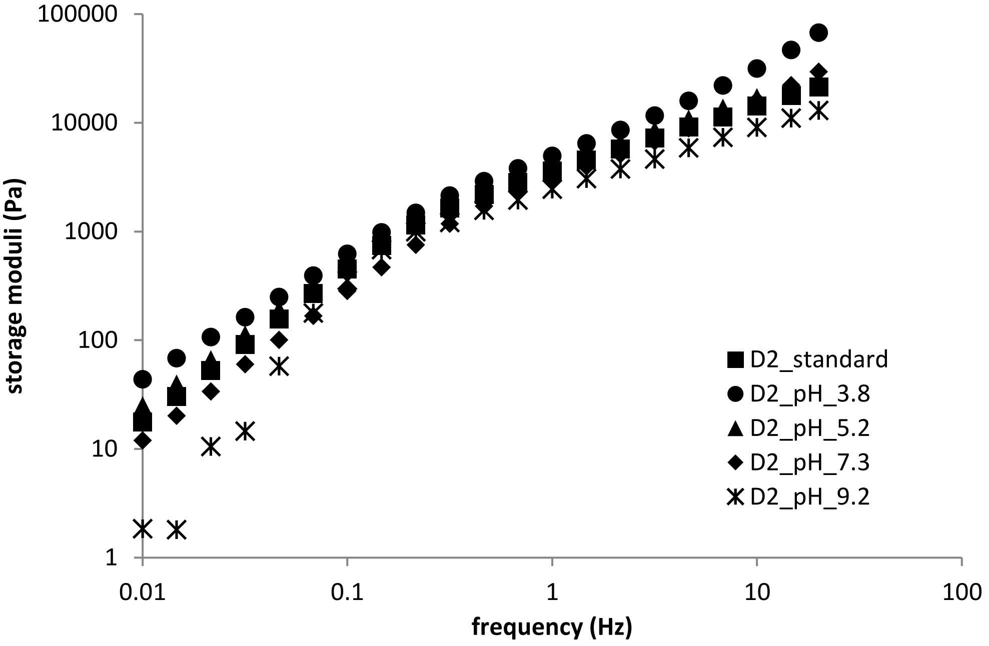

Figure S12. Storage moduli (G′) for D2 sample – pH dependence

**Sample**	**Mesh size (nm)**			
	**original sample**	**$CaCl_2$**	**$MgCl_2 \cdot 6H_2O$**	**$FeCl_3$**
**H1**	23.23	17.80	16.57	16.01
**H2**	24.55	15.82	15.93	20.55
**H3**	23.01	15.23	17.64	21.54
**H4**	39.00	22.39	22.08	25.98
**H5**	25.62	16.93	21.91	19.43
**H6**	27.77	15.40	19.43	23.17
**D1**	8.94	7.68	6.55	6.22
**D2**	7.12	-	9.29	13.82

Table S7. Calculated values for the mesh size of phase-separated hydrogels. Describe the effect of multivalent ions to mesh size in comparison with mesh size of original samples

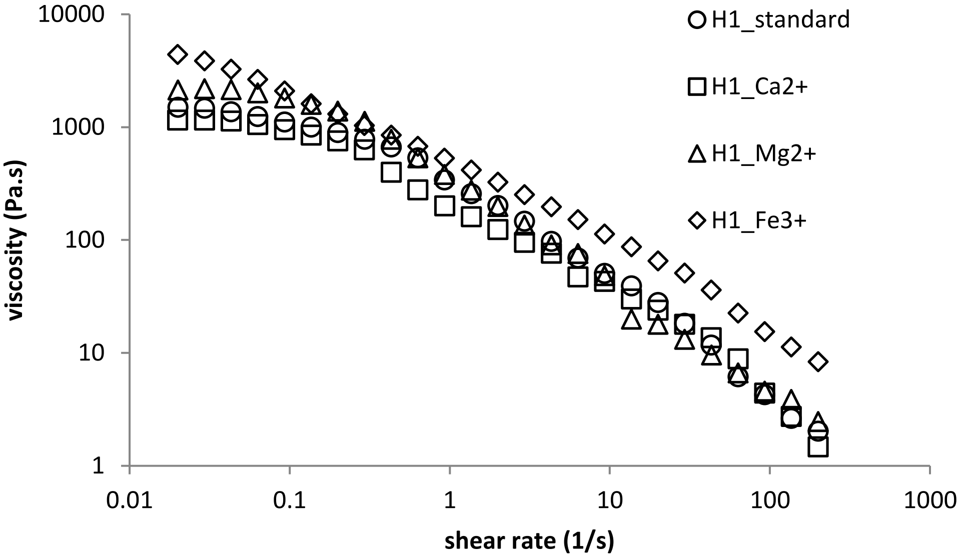

Figure S13. Flow properties of H1 sample – the effect of multivalent ions

72

73

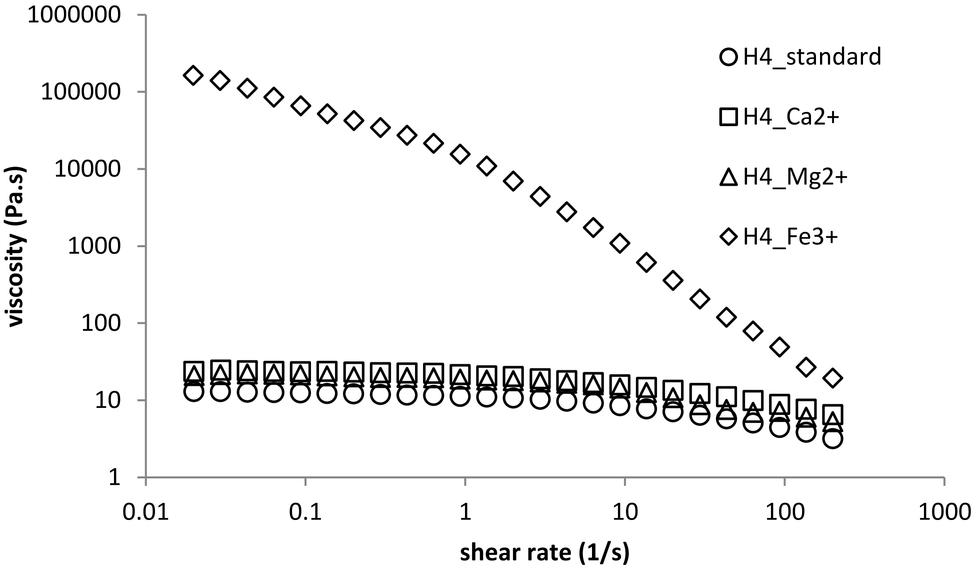

Figure S14. Flow properties of H4 sample – the effect of multivalent ions

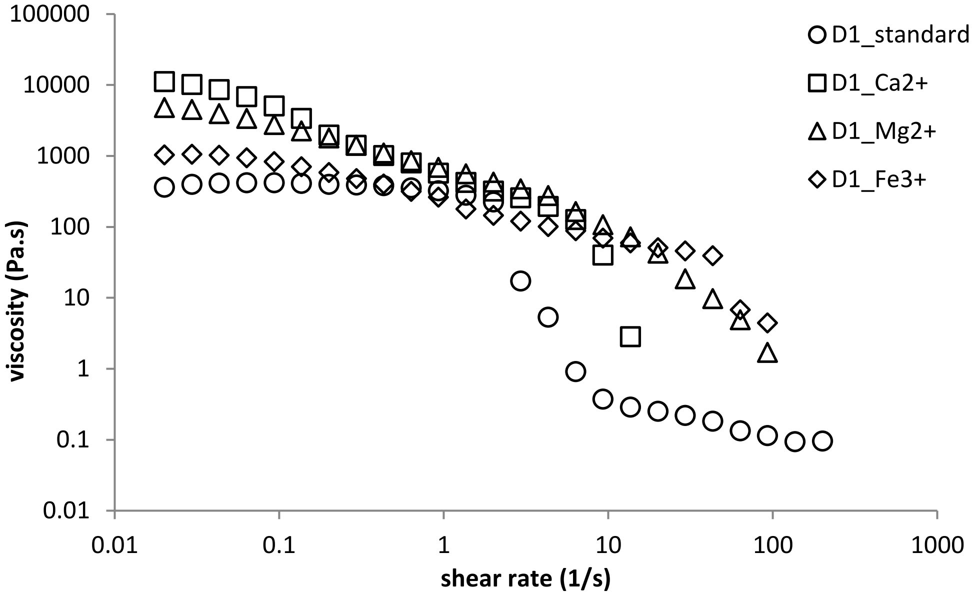

Figure S15. Flow properties of D1 sample – the effect of multivalent ions

78

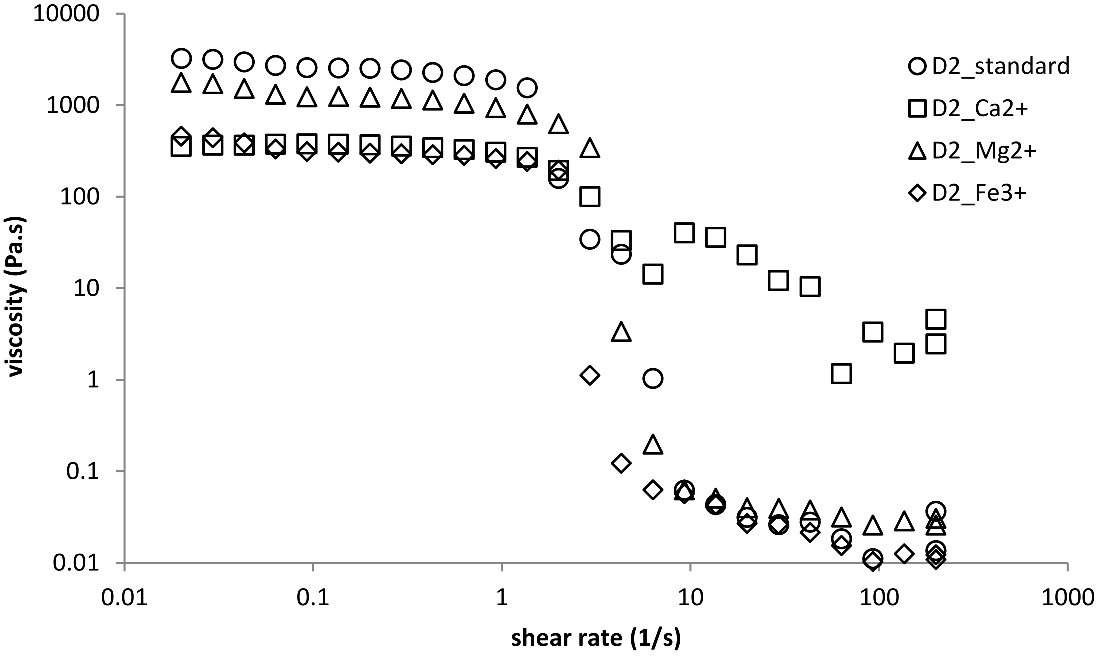

Figure S16. Flow properties of D2 sample – the effect of multivalent ions

79
